# The Impact of Assisted Dying Legislation on Nursing Practice in Palliative Care: A Scoping Review

**DOI:** 10.1111/jan.70657

**Published:** 2026-06-05

**Authors:** Marlene Werner, Christiane Kreyer, Sabine Pleschberger

**Affiliations:** ^1^ Department of Primary Care Medicine, Centre for Public Health Medical University of Vienna Vienna Austria; ^2^ Department of Nursing Science, Faculty of Social Sciences University of Vienna Vienna Austria; ^3^ Department of Nursing Science and Gerontology, Institute of Nursing Science UMIT TIROL—Private University for Health Sciences and Technology Hall in Tirol Austria

**Keywords:** assisted dying, nursing, palliative care, review

## Abstract

**Aim:**

To review the literature on the state of research on the impacts of assisted dying on nursing practice within specialised palliative care.

**Design:**

A scoping review was conducted in accordance with the Joanna Briggs Institute methodology for scoping reviews.

**Data Sources:**

PubMed, Embase, CINAHL, PsycINFO and CENTRAL were searched between July and August 2024.

**Methods:**

Articles were included if they referred to countries in which assisted dying is legally permitted and is understood as a practice that is not aligned with the philosophy of palliative care, enabling analysis of its impact on nursing practice in specialised palliative care. After the screening process, data were extracted and then synthesised using thematic analysis.

**Results:**

Fifteen studies published between 2019 and 2024, all from Canada or the United States, met the inclusion criteria. Three themes were identified: (1) positioning and meaning, describing how nurses are required to position themselves and to renegotiate their values; (2) impact on core competencies, capturing changes in key nursing responsibilities; and (3) challenges in interpersonal relationships, referring to increased team conflicts and shifts in relationships with patients and their families.

**Conclusion:**

The legalisation of assisted dying impacts nursing practice in palliative care in various ways, challenging the established advanced practice role of specialist palliative care nurses. This calls for comprehensive ethical reflection within the nursing profession regarding its role and core values in this context.

**Impact:**

This review identifies significant challenges facing advanced nursing roles and the palliative care discipline. It provides a foundation for future research and ethical deliberation, with relevance for nurses, educators, policymakers and researchers involved in end‐of‐life care.

**Patient or Public Contribution:**

This study did not include patient or public involvement in its design, conduct or reporting.

## 1 Introduction

An increasing number of countries have introduced legal frameworks for assisted dying (AD) in the past years (Mroz et al. 2021). In this paper, ‘AD’ will be used as an umbrella term encompassing the legal frameworks related to euthanasia and assisted suicide. These practices differ in terms of who administers the life‐ending medication: in assisted suicide, the individual self‐administers the lethal dose, whereas in euthanasia, it is administered by a healthcare professional (Radbruch et al. 2016). Although laws on AD differ in terms of eligibility criteria and procedural requirements, they are based on a common rationale, namely the widely held belief that individuals facing certain terminal illnesses should have the legal option to hasten death in order to preserve their autonomy and dignity at the end of life (Sallnow et al. 2022).

However, the desire to reduce suffering and preserve dignity at the end of life is not new. The field of palliative care emerged precisely in response to the inadequate and often distressing conditions under which people were dying in hospitals (Saunders 2001). Palliative care takes a holistic and multidisciplinary approach to improving the quality of life of patients and their families facing life‐threatening illness (WHO 2002). Its focus lies in relieving pain and other distressing symptoms, but with the aim to ‘neither hastening nor postponing death’ as one of its key principles (WHO 2002). Leading organisations such as the European Association for Palliative Care (EAPC) and the International Hospice and Palliative Care Association (IHPCA) have explicitly stated that AD is incompatible with the principles of palliative care and should remain separate from its practice (De Lima et al. 2017; Radbruch et al. 2016).

Nonetheless, a review by Gerson, Koksvik, et al. (2020) shows that the relationship between AD and palliative care varies widely across geographical contexts, with Belgium and the Netherlands representing unique cases, as both are characterised by a palliative care philosophy in which AD is not primarily understood as a mutually exclusive approach. In the Netherlands, palliative care developed in a context where AD had already become an established practice (Gerson, Koksvik, et al. 2020), whereas in Belgium, palliative care pioneers actively supported the legalisation of AD and its inclusion as an additional option within palliative care (Bernheim et al. 2008). However, in other countries, the introduction of AD legislation is usually accompanied by questions regarding its compatibility with palliative care, leading to ethical challenges and moral questions regarding two conflicting end‐of‐life options (Inbadas et al. 2020). This, in turn, leads to uncertainties for palliative care professionals regarding their role (Gerson, Koksvik, et al. 2020), an issue which is strongly associated with moral distress, particularly among nurses (Klotz et al. 2025).

By definition, nurses play a central role in palliative care (WHO 2002). In many countries, advanced practice roles have emerged and this involves specialised training in symptom management, end‐of‐life communication, grief support and the ethical and legal aspects of dying (Ferrell and Paice 2015). The ethical framework of palliative care nurses is shaped not only by the philosophy of palliative care but also by the Code of Ethics of the International Council of Nurses (2021). This code articulates core professional values and responsibilities, including the promotion of a dignified death, justice, advocacy, responsiveness, compassion and trustworthiness. With the legalisation of AD, nurses face the particularly challenging task of reconciling implications of this practice with the core values of palliative care and nursing ethics.

A review focusing on the implications of AD for nursing highlights that the extent of nurses' involvement in the process remains largely unclear and that appropriate policies or professional guidelines are often lacking (Richardson 2023). Nevertheless, research from Belgium shows that nurses are involved in various ways, most frequently when receiving requests for AD (Bellon et al. 2022). Nurses experience supporting such requests as emotionally and ethically challenging and feel that they are not adequately prepared for it (Sandham et al. 2022). Although nurses have the right to opt out, studies show that exercising conscientious objection can be difficult in practice (Lamb et al. 2018). Attitudes towards AD were found to be mixed, with perspectives being shaped by personal, professional and relational factors (Cayetano‐Penman et al. 2020). In summary, there is a growing body of research on nurses' experiences, roles and attitudes in relation to AD. However, there is still a notable gap regarding its concrete impact on nursing practice, particularly when moving beyond the question of whether nurses are directly involved in specific AD practices. Due to their expertise, values and holistic approach, the perspectives of palliative care nurses can provide valuable insights into the broader ethical and practical challenges that arise in end‐of‐life care within the context of legalised AD.

Therefore, this review focuses on nurses in specialised palliative care settings in countries where AD is not considered part of hospice or palliative care practice.

## 2 The Aim

This study aims to review the literature on the state of research on the impacts of AD on nursing practice within specialised palliative care.

## 3 Methods

A scoping review was conducted in accordance with the Joanna Briggs Institute methodology (Peters et al. 2020). Scoping reviews aim to systematically map available evidence, identify key concepts and highlight knowledge gaps (Arksey and O'Malley 2005). Given the variety of terms and definitions surrounding AD, palliative care and nursing roles, this methodology was deemed the most suitable approach to map heterogeneous evidence, explore how research has been conducted and clarify how definitions are used in the literature (Munn et al. 2018). A protocol was developed to support planning and ensure methodological quality, based on predefined objectives, research questions and methods. We follow the PRISMA‐ScR guidance for reporting scoping reviews in this paper (Tricco et al. 2018).

### 3.1 Search Methods

A structured database search was developed in collaboration with the library services of the Medical University of Vienna. The search strategy combined MeSH terms and keywords covering three core concepts: *assisted dying*, *nurses* and *palliative care*. Search strings were adapted for each database and designed to retrieve studies where the relevant terms appeared in the title or abstract. To ensure alignment with the research question, trial searches were conducted and evaluated through preliminary screening. No restrictions were applied to the publication date. The following databases were searched: PubMed, Embase, CINAHL, PsycINFO and CENTRAL. The final search was carried out between July and August 2024. Exact search dates and full search strings are provided in Supporting Information S1. We used software such as EndNote 20 and Rayyan to organise and manage the search results.

### 3.2 Inclusion Criteria

#### 3.2.1 Participants

Studies were eligible for inclusion if specialised palliative care nurses were part of the study sample, regardless of participants' age, culture, ethnicity, gender or level of experience. Given the multiprofessional nature of palliative care, nurses had to be clearly identified as a distinct subgroup within the sample. Studies were excluded if their findings did not sufficiently focus on nurses or could not be clearly attributed to palliative care contexts.

#### 3.2.2 Concept

Our review focused on the implications of AD for nursing practice in specialised palliative care, not on the practice of AD itself. Studies exclusively addressing AD procedures, other end‐of‐life practices (e.g., palliative sedation) or nurses' personal attitudes and beliefs about AD were excluded.

#### 3.2.3 Context

We considered all geographical areas in which AD is legally permitted and is understood as a practice that is not aligned with the philosophy of palliative care. Studies from the Netherlands and Belgium were excluded, as AD and palliative care are conceptually and institutionally intertwined in these contexts, which limits the ability to analyse the impact of the legalisation of AD on nursing practice in specialised palliative care.

#### 3.2.4 Types of Sources

This scoping review included only peer‐reviewed primary research studies. Eligible study designs comprised qualitative, quantitative and mixed‐methods approaches. Only publications written in English or German were considered.

### 3.3 Search Outcome

The initial search yielded 2257 records. After duplicates were removed, 1390 records remained for screening. The screening was conducted in two stages: (1) Titles and abstracts were screened independently by two reviewers (M.W. and S.P.) and categorised as *included*, *maybe* or *excluded* based on the inclusion criteria. This led to the exclusion of 1301 papers. (2) Full‐text screening of the remaining 89 records was conducted by the same reviewers. Two full texts could not be retrieved. One was inaccessible due to paywall restrictions, and one referred to a non‐retrievable article due to an indexing error in PubMed. 71 sources were excluded for various reasons, including inappropriate study types (e.g., editorials or opinion papers), publication prior to the enactment of assisted dying legislation or an incorrect study focus (e.g., palliative sedation or absence of a clearly identifiable nursing perspective). Discrepancies were resolved through discussion, where no consensus could be reached, a third researcher (C.K.) was consulted. Details of the selection process are illustrated in Figure 1.

**FIGURE 1:**
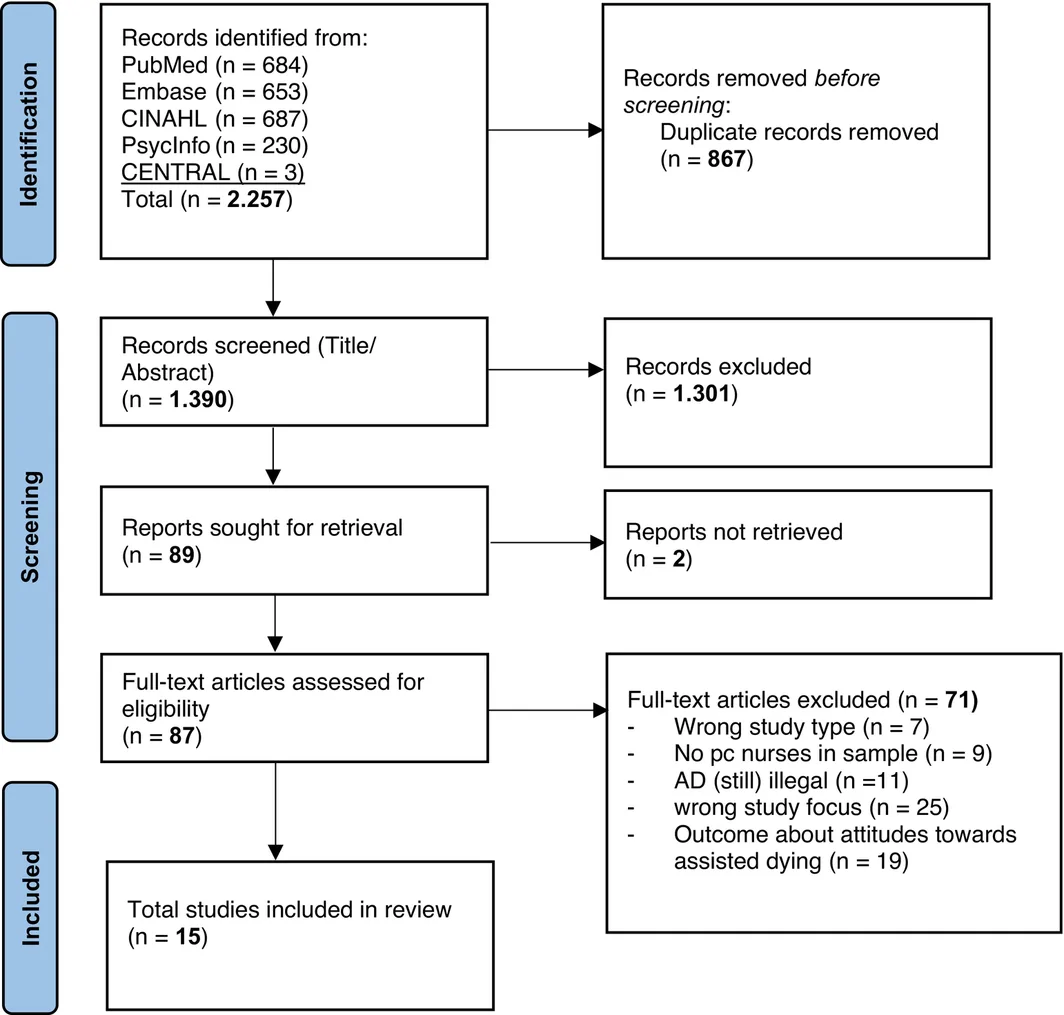
PRISMA flowchart.

### 3.4 Data Abstraction and Synthesis

Data extraction was carried out by the first author (M.W.), who compiled key information from the included studies into a structured summary and a data extraction table. This table presents details such as authorship, year of publication, country, AD legislation, research objective, study design, sample and setting (see Table 1).

**TABLE 1:** Study characteristics.

Authors, year	Country	AD legislation	Objective	Study design	Sample	Setting
Antonacci et al. (2021)	Ca	Medical Assistance in Dying (MAiD): legalised euthanasia and assisted suicide in 2016	Members of the Canadian Hospice Palliative Care Association (CHPCA) on integrating MAiD into end‐of‐life care and supporting healthcare providers involved in MAiD	Quantitative study/online survey	452 members of CHPCA: palliative care nurses (*n* = 161), administrators (*n* = 79), family caregiver (*n* = 6), personal support worker (*n* = 9), physician (*n* = 56), spiritual care provider (*n* = 16), volunteer (*n* = 76), other (*n* = 89)	Residential hospices, long‐term care, home care, acute care, prison, palliative care society, prison, shelter, other
Buchbinder et al. (2019)	USA—Vermont	Patient Choice and Control at End‐of‐Life Act (Act 39): legalised assisted suicide in 2013	Experiences of Vermont healthcare providers in implementing Act 39 and participating in the medical aid‐in‐dying process	Qualitative study/semi‐structured interviews	37 health care providers: palliative care nurses (*n* = 11), physicians (*n* = 19), social worker (*n* = 18), nurses (*n* = 16)	Family medicine, hospice/palliative care, neurology, oncology, hospital based, home care
Freeman et al. (2021)	Ca	MAiD	Impact of MAiD legalisation on hospice care provider roles in non‐provider contexts	Qualitative study/semi‐structured interviews	Same as Ward et al. (2022): 8 hospice and palliative care providers: no further information but sample includes hospice nurses	Hospice facility that did not permit MAiD procedures
Gerson, Preston, et al. (2020)	USA—Washington State	Death with Dignity Act: legalised assisted suicide in 2009	Hospice professionals' experiences with patients who hasten death or die by suicide in Washington State, with or without legal assistance	Qualitative study/semi‐structured interviews	21 hospice and palliative care providers: palliative care nurses (*n* = 7), social workers (*n* = 7), physicians (*n* = 4) and chaplains (*n* = 3)	From different types of hospice organisations (religious/nonreligious/profit/non‐profit) primarly provide services in home settings
Guité‐Verret et al. (2024)	Ca	MAiD	Clinicians' experiences with continuous sedation and the influence of assisted death on palliative sedation practices	Qualitative study/semi‐structured focus groups	28 hospice and palliative care providers: palliative care nurses (*n* = 15), physicians (*n* = 12), end of life doula (*n* = 1)	Inpatient hospice, community, inpatient care, long‐term care, retirement home
Ho, Joolaee, et al. (2021)	Ca	MAiD	Exploration of challenges and resource recommendations for palliative and hospice care providers caring for patients considering MAiD	Qualitative study/semi‐structured interviews	26 hospice and palliative care providers: palliative care nurses (*n* = 12), social workers (*n* = 5), physicians (*n* = 7), spiritual health practitioners (*n* = 2)	Hospice, hospital palliative care, community, multiple sites
Ho, Norman, et al. (2021)	Ca	MAiD	Impact of MAiD legislation on end‐of‐life care discussions between patients and palliative care providers	Qualitative study/semi‐structured interviews	Same as Joolaee et al. (2022): 48 hospice and palliative care providers: palliative care nurses (*n* = 15), physicians (*n* = 22), social worker (*n* = 7), allied health professional (*n* = 4)	Hospice, hospital palliative care, community, multiple sites
Joolaee et al. (2022)	Ca	MAiD	Positive aspects of MAiD legalisation from hospice palliative care providers' perspectives	Qualitative study/semi‐structured interviews	Same as Ho, Norman, et al. (2021): 48 hospice and palliative care providers: palliative care nurses (*n* = 15), physicians (*n* = 22), social worker (*n* = 7), allied health professional (*n* = 4)	Hospice, hospital palliative care, community, multiple sites
Mathews et al. (2021)	Ca	MAiD	Frontline palliative care providers' experiences with the impact of MAiD on practice	Qualitative study/semi‐structured interviews	23 hospice and palliative care providers: palliative care nurses (*n* = 10) and palliative care physicians (*n* = 13)	Inpatient consult services, inpatient palliative care units, outpatient clinics, home‐based palliative care and residential hospices
Pesut, Thorne, Schiller, Greig, and Roussel (2020)	Ca	MAiD	Understanding the implications of legislated assisted death on nurses' experiences and nursing practice	Qualitative study/semi‐structured interviews	Same as Pesut, Thorne, Schiller, Greig, and Roussel (2020): 59 nurses from diverse settings: palliative care nurses (*n* = 4)	Home and community (*n* = 32), acute care (*n* = 10), long term care (*n* = 5), hospice (*n* = 4), clinic (*n* = 3) and other care settings (*n* = 8)
Pesut, Thorne, Schiller, Greig, Roussel, and Tishelman (2020)	Ca	MAiD	Exploration of how nurses define good nursing practice in the context of MAiD	Qualitative study/semi‐structured interviews	Same as Pesut, Thorne, Schiller, Greig, Roussel, and Tishelman (2020): 59 nurses from diverse settings: palliative care nurses (*n* = 4)	Home and community (*n* = 32), acute care (*n* = 10), long term care (*n* = 5), hospice (*n* = 4), clinic (*n* = 3) and other care settings (*n* = 8)
Pesut, Thorne, et al. (2021)	Ca	MAiD	Nurses' experiences with MAiD legislation changes from Bill C‐14 to Bill C‐7, including eligibility and waiting period changes	Qualitative study/semi‐structured interviews	Same as Pesut, Wright, et al. (2021): 50 nurses from diverse settings: palliative care nurses (*n* = 26)	Specialised palliative setting (*n* = 26), primary care (*n* = 17), medical surgical (*n* = 13) and other settings (*n* = 11)
Pesut, Wright, et al. (2021)	Ca	MAiD	Nurses' perspectives on the understanding and application of suffering in MAiD	Qualitative study/semi‐structured interviews and document review	Same as Pesut, Thorne, et al. (2021): 50 nurses from diverse settings: palliative care nurses (*n* = 26)	Specialised palliative setting (*n* = 26), primary care (*n* = 17), medical surgical (*n* = 13) and other settings (*n* = 11)
Ward et al. (2022)	Ca	MAiD	Hospice care providers' perceptions of MAiD in a facility that does not provide MAiD	Qualitative study/semi‐structured interviews	Same as Freeman et al. (2021): 8 hospice and palliative care providers: no further information but sample includes hospice nurses	Hospice facility that did not permit MAiD procedures
Wright et al. (2021)	Ca	MAiD	How palliative care nurses negotiate their moral identity in relation to MAiD, and the tension with the narrative excluding MAiD	Qualitative study/semi‐structured interviews	Palliative care nurses (*n* = 22)	Home care, acute care, long‐term care, hospital palliative care units and hospices

A thematic analysis was conducted following the approach of Braun and Clarke (2022). One author (M.W.) systematically reviewed the included texts to extract recurring ideas, concepts and patterns. Relevant textual data were inductively coded using MAXQDA 2024. As an initial step in the analysis, a random sample of four studies was independently reviewed and discussed with a third author (S.P.) to support the development and refinement of the coding framework and to ensure analytic consistency. Through this process, extracted data were abstracted into broader themes, which were iteratively discussed to ensure analytical coherence and depth.

As some included studies used multiprofessional samples, we paid particular attention to identifying nursing‐specific perspectives within these data. In most studies, findings were presented in a way that allowed profession‐specific perspectives to be clearly distinguished (e.g., through separate reporting of nurses' quotations or dedicated sections in the discussion). In some studies, this distinction was less explicit, as results were presented as shared experiences within multiprofessional teams. However, if these results were obviously informed by nursing perspectives, were considered them as relevant for nursing practice.

## 4 Results

### 4.1 Summary of Study Characteristics

A total of 15 studies met the inclusion criteria for this review (see Table 1). All were published between 2019 and 2024 and conducted either in Canada (*n* = 13) or the USA (*n* = 2). The legal context of these studies differs as follows: In Canada, Medical Assistance in Dying (MAiD) was legalised in 2016, allowing euthanasia and assisted suicide. In the USA, laws differ at state level. One study is from Vermont, where the Patient Choice and Control at End‐of‐Life Act was legalised in 2013, permitting assisted suicide (Buchbinder et al. 2019). Another study is located in Washington State, where the Death with Dignity Act was legalised in 2009, also permitting assisted suicide (Gerson, Preston, et al. 2020). In the following sections, we use terms associated with national frameworks when specifically referring to a respective legal context (e.g., in direct quotations) and otherwise stick to the overarching term AD, as explained at the beginning.

Only one study was based on a sample with palliative care nurses exclusively (Wright et al. 2021). All others involved either multiprofessional teams of hospice and palliative care professionals (e.g., nurses, physicians, social workers) or nurses from diverse settings and disciplines, including palliative care.

All studies were conducted in hospices or specialised palliative care settings. Some of the studies also recruited participants from other settings, including long‐term care (*n* = 5), home care (*n* = 5) or acute care settings (*n* = 4). Two studies were explicitly conducted in so‐called ‘non‐provider’ contexts where AD is prohibited (Freeman et al. 2021; Ward et al. 2022).

The majority of the studies (*n* = 13) applied qualitative semi‐structured interviews for data collection. Other methods included focus groups (*n* = 1), document review (*n* = 1) and online surveys (*n* = 1).

Some studies were based on overlapping data sets, resulting in a total of 11 distinct samples (see ‘sample’ column in Table 1).

### 4.2 Identified Themes From Scoping Review

A total of nine subthemes were identified and grouped into three main themes: (1) positioning and meaning, (2) impacts on core competencies and (3) challenges in interpersonal relationships. Table 2 provides an overview of the individual studies and their contributions to the identified subthemes.

**TABLE 2:** Overview of individual studies and their contributions to the (sub)themes.

Authors, year	Positioning & meaning			Impact on core competencies			Challenges in interpersonal relationships		
	The necessity of taking a stand	Changing images of suffering death and dying	Reputation of palliative care within society	Conversations become more difficult	Symptom management	Emerging tasks	Patient‐ provider relationship	Interacting with relatives	Collaboration
Antonacci et al. (2021)	x	x	x				x	x	x
Buchbinder et al. (2019)	x		x	x		x			x
Freeman et al. (2021)	x	x	x	x	x	x	x	x	x
Gerson, Preston, et al. (2020)				x		x			x
Guité‐Verret et al. (2024)	x		x	x		x		x	
Ho, Joolaee, et al. (2021)	x	x	x	x	x		x	x	x
Ho, Norman, et al. (2021)	x	x	x	x	x	x	x	x	x
Joolaee et al. (2022)			x			x			x
Mathews et al. (2021)	x	x	x	x	x	x	x	x	x
Pesut, Thorne, Schiller, Greig, and Roussel (2020)	x		x	x		x			x
Pesut, Thorne, Schiller, Greig, Roussel, and Tishelman (2020)	x	x		x		x	x		
Pesut, Thorne, et al. (2021)			x	x		x		x	x
Pesut, Wright, et al. (2021)		x		x	x	x		x	
Ward et al. (2022)	x	x		x	x	x	x		x
Wright et al. (2021)	x	x	x	x	x		x		x

#### 4.2.1 Positioning and Meaning

Nurses need to reflect on their individual position regarding AD, consider its implications for their approach to the dying process and ultimately understand its broader impact on nursing in palliative care within a societal context.

**The necessity of taking a stand.** In nearly all included studies, the struggle of reflecting and taking a stand towards AD was described as a process of moral deliberation (Antonacci et al. 2021; Buchbinder et al. 2019; Freeman et al. 2021; Guité‐Verret et al. 2024; Ho, Joolaee, et al. 2021; Mathews et al. 2021; Pesut, Thorne, Schiller, Greig, and Roussel 2020; Pesut, Thorne, Schiller, Greig, Roussel, and Tishelman 2020) or referred to as ‘soul‐searching’ (Wright et al. 2021). Taking a stand is described as emotionally intense because it may lead to questioning, potentially rethinking core values (Antonacci et al. 2021; Buchbinder et al. 2019; Ho, Joolaee, et al. 2021; Mathews et al. 2021; Pesut, Thorne, Schiller, Greig, and Roussel 2020; Pesut, Thorne, Schiller, Greig, Roussel, and Tishelman 2020; Ward et al. 2022; Wright et al. 2021). As a result, the reported experiences include feelings of confusion, inner conflict and uncertainty (Antonacci et al. 2021; Freeman et al. 2021; Ho, Joolaee, et al. 2021; Ward et al. 2022; Wright et al. 2021).

Studies indicate that nurses perceive not only finding one's position on AD can be challenging, but also articulating and communicating this stance to others. Depending on the dominant view within a team, speaking up can lead to disagreement or challenging situations (Freeman et al. 2021; Mathews et al. 2021; Ward et al. 2022; Wright et al. 2021). Wright et al. (2021) even refer to this process as a form of ‘outing’.

Even though several studies report that nurses perceive tensions arising from conflicting views on AD, they also describe a shared professional commitment: staying with the patient and maintaining a caring relationship (Buchbinder et al. 2019; Ho, Norman, et al. 2021; Pesut, Thorne, Schiller, Greig, and Roussel 2020; Pesut, Thorne, Schiller, Greig, Roussel, and Tishelman 2020; Wright et al. 2021). Furthermore, two studies indicate that nurses who object to AD still wish to remain involved in patients' care (Pesut, Thorne, Schiller, Greig, Roussel, and Tishelman 2020; Wright et al. 2021).

##### 4.2.1.1 Changing Images of Suffering, Death and Dying

Following the legalisation of AD, palliative care nurses reflect on how AD changes traditional images of death and dying. A frequently mentioned aspect is the accelerated pace of dying, which challenges the understanding of the nature of dying within palliative care (Freeman et al. 2021; Ho, Norman, et al. 2021; Mathews et al. 2021; Pesut, Thorne, Schiller, Greig, Roussel, and Tishelman 2020; Pesut, Wright, et al. 2021; Ward et al. 2022; Wright et al. 2021). From the nurses' perspective, AD enables patients to set a specific date for death, allowing them to plan and orchestrate their dying process (Pesut, Thorne, Schiller, Greig, Roussel, and Tishelman 2020; Pesut, Wright, et al. 2021). Furthermore, nurses understand suffering in the context of AD as existential distress, particularly driven by the fear of losing autonomy and control and less about physical symptoms (Ho, Joolaee, et al. 2021; Ho, Norman, et al. 2021; Pesut, Wright, et al. 2021).

Nurses emphasise the different ways of dying by using specific terms or metaphors such as ‘different’ (Freeman et al. 2021; Ho, Joolaee, et al. 2021; Pesut, Thorne, Schiller, Greig, and Roussel 2020; Pesut, Thorne, Schiller, Greig, Roussel, and Tishelman 2020), ‘creepy’ (Pesut, Thorne, Schiller, Greig, Roussel, and Tishelman 2020), ‘artificial’ (Freeman et al. 2021), a ‘Caesarean death’ (Pesut, Thorne, Schiller, Greig, Roussel, and Tishelman 2020) and as an ‘event’ (Pesut, Wright, et al. 2021). They consider AD as a way to shorten or avoid the terminal phase of life (Mathews et al. 2021; Pesut, Thorne, Schiller, Greig, Roussel, and Tishelman 2020; Pesut, Wright, et al. 2021; Ward et al. 2022; Wright et al. 2021). Consequently, they see AD as a shift away from the traditional palliative care focus on supporting quality of life throughout the dying process (Antonacci et al. 2021; Freeman et al. 2021; Ho, Norman, et al. 2021; Pesut, Thorne, Schiller, Greig, Roussel, and Tishelman 2020; Ward et al. 2022). This perceived shift is illustrated by nurses telling stories about patients requesting AD even when they were far from dying (Freeman et al. 2021; Pesut, Thorne, Schiller, Greig, Roussel, and Tishelman 2020; Ward et al. 2022).

##### 4.2.1.2 Reputation of Palliative Care Within Society

At the societal level, palliative care has long faced limited visibility and persistent misconceptions about its goals and practices: ‘Others […] worrying that PCPs' [palliative care professionals] involvement in MAiD may further blur the public perception of palliative care and erode trust in a specialty that commits to whole‐person care by easing pain and suffering without hastening death’ (Ho, Norman, et al. 2021, 7). Across studies participants raise concerns that the growing public debate around AD may result in palliative care being overshadowed and a shift in end‐of‐life priorities (Ho, Joolaee, et al. 2021; Pesut, Thorne, Schiller, Greig, and Roussel 2020; Wright et al. 2021). In contrast, they recognise that this debate has also raised awareness of advance care planning and contributed to greater recognition of palliative care (Guité‐Verret et al. 2024; Joolaee et al. 2022; Mathews et al. 2021; Pesut, Thorne, et al. 2021).

However, studies report that both the public and healthcare professionals often struggle to clearly distinguish between palliative care and AD, which contributes to confusion and uncertainty (Antonacci et al. 2021; Buchbinder et al. 2019; Guité‐Verret et al. 2024; Ho, Norman, et al. 2021). Furthermore, many studies report palliative care nurses' concerns about resource allocation, particularly as they see new responsibilities being added to already limited palliative and hospice resources (Antonacci et al. 2021; Freeman et al. 2021; Ho, Joolaee, et al. 2021; Ho, Norman, et al. 2021; Mathews et al. 2021; Pesut, Thorne, Schiller, Greig, and Roussel 2020; Pesut, Thorne, et al. 2021; Wright et al. 2021).

#### 4.2.2 Impacts on Core Competencies

Studies indicate that nurses perceive a noticeable shift in how holistic palliative care is being provided in the light of AD. On the one hand, nurses report that AD can restrict or complicate nurses' ability to utilise their full range of competencies, for example in conducting conversation or symptom control. On the other hand, AD reshapes their professional roles and scope of practice, with some tasks gaining prominence and others being newly introduced.

##### 4.2.2.1 Conversations Become More Difficult

Although palliative care nurses are highly skilled in communication and have long been familiar with requests to die, studies report that nurses perceive conversations to become more difficult with the introduction of AD (Freeman et al. 2021; Gerson, Preston, et al. 2020; Ho, Joolaee, et al. 2021; Ho, Norman, et al. 2021; Mathews et al. 2021; Pesut, Thorne, Schiller, Greig, Roussel, and Tishelman 2020; Pesut, Thorne, et al. 2021; Ward et al. 2022; Wright et al. 2021).

Nurses report that uncertainty and hesitation can impair communication, hinder symptom management and reduce the overall support provided to patients (Freeman et al. 2021; Ho, Norman, et al. 2021; Pesut, Thorne, Schiller, Greig, Roussel, and Tishelman 2020). Three studies have reported that time constraints in daily work can restrict conversations about the underlying motivations behind AD requests (Pesut, Thorne, Schiller, Greig, and Roussel 2020; Pesut, Wright, et al. 2021; Ward et al. 2022). In addition to limited time resources, difficulties in finding appropriate language are also commonly described and can further complicate communication (Freeman et al. 2021; Ward et al. 2022; Wright et al. 2021). Questions related to whether nurses should proactively address the topic of AD further contribute to confusion and insecurity (Ho, Norman, et al. 2021; Mathews et al. 2021; Pesut, Thorne, Schiller, Greig, and Roussel 2020). Several studies have highlighted that institutional policies may restrict open conversations or discourage staff from engaging in discussions that could influence decisions (Freeman et al. 2021; Gerson, Preston, et al. 2020; Ho, Norman, et al. 2021; Wright et al. 2021). In a few cases, this has resulted in AD decisions being made without consulting palliative care professionals (Freeman et al. 2021; Ho, Joolaee, et al. 2021; Ho, Norman, et al. 2021; Pesut, Thorne, Schiller, Greig, Roussel, and Tishelman 2020; Pesut, Thorne, et al. 2021).

Conversely, some studies report that discussing AD as an additional option at the end of life enables open dialogues about death and dying (Buchbinder et al. 2019; Gerson, Preston, et al. 2020; Guité‐Verret et al. 2024; Joolaee et al. 2022; Pesut, Thorne, et al. 2021) and ‘some participants felt positive about providing patients a sense of control by discussing MAiD as part of end‐of‐life care planning’ (Ho, Norman, et al. 2021, 6).

##### 4.2.2.2 Symptom Management

Authors analysed several reported situations in which palliative care nurses encountered patients who refused symptom‐relieving interventions, despite ongoing suffering (Ho, Joolaee, et al. 2021; Ho, Norman, et al. 2021; Mathews et al. 2021; Ward et al. 2022). These refusals were often linked to patients' fears of becoming ineligible for AD due to the potential sedating effects of symptom management (Ho, Joolaee, et al. 2021; Ho, Norman, et al. 2021; Mathews et al. 2021). Nurses interpret that the symptoms of existential distress outweigh physical pain and that maintaining autonomy is more important for patients (Ho, Joolaee, et al. 2021; Ho, Norman, et al. 2021; Pesut, Wright, et al. 2021). Nurses report that this creates emotional tension because they are unable to provide effective symptom management, which they consider to be a core element of their discipline (Freeman et al. 2021; Ho, Norman, et al. 2021; Ward et al. 2022; Wright et al. 2021).

##### 4.2.2.3 Emerging Tasks

Regardless of nurses' stance or involvement in AD, studies indicate changes in the scope of nursing practice on multiple levels. For example, clarifying a patient's wish to die has long been part of palliative care. However, with the legal option of AD, nurses reported perceiving this task as more urgent and important, as it may lead to a formal request for AD (Pesut, Thorne, Schiller, Greig, Roussel, and Tishelman 2020; Pesut, Wright, et al. 2021; Ward et al. 2022). More information is needed in the context of AD regarding who to contact when a request is made (Buchbinder et al. 2019; Freeman et al. 2021; Joolaee et al. 2022; Ward et al. 2022) or where to access AD medications (Gerson, Preston, et al. 2020). Nurses in the study by Gerson, Preston, et al. (2020, 682) described feeling unable to completely avoid involvement with AD: ‘Participants suggested that despite organisational policies, MAID cannot be ignored in their effort to provide care. RN7 highlights how some patients do not involve hospice professionals as they are going through the process of obtaining MAID medications but then call for assistance after the death.’

Furthermore, two studies demonstrate that professionals from other disciplines and care settings often rely on palliative care professionals for guidance about AD (Pesut, Thorne, Schiller, Greig, Roussel, and Tishelman 2020; Pesut, Thorne, et al. 2021). This appears to be linked to the specific expertise of palliative care nurses in managing suffering and exploring options, which is considered critical in the context of AD (Ho, Norman, et al. 2021; Pesut, Thorne, Schiller, Greig, and Roussel 2020; Pesut, Thorne, Schiller, Greig, Roussel, and Tishelman 2020; Pesut, Thorne, et al. 2021). It has also been reported that palliative care professionals in particular often play a key role in shaping AD policy at service levels (Joolaee et al. 2022; Pesut, Thorne, Schiller, Greig, and Roussel 2020; Pesut, Thorne, Schiller, Greig, Roussel, and Tishelman 2020; Pesut, Thorne, et al. 2021). In addition, Guité‐Verret et al. (2024) and Mathews et al. (2021) point out that nurses feel responsible for educating colleagues, patients and families on the differences between palliative care interventions and AD.

#### 4.2.3 Challenges in Interpersonal Relationships

Given that person‐centred care, family involvement and multidisciplinary teamwork are core principles of palliative care, it is unsurprising that legislation on AD has implications for relationships at all levels.

##### 4.2.3.1 Patient‐Provider Relationship

As demonstrated above communication gaps related to AD can strain nurse–patient relationships, as reported in several studies (Freeman et al. 2021; Ho, Joolaee, et al. 2021; Ho, Norman, et al. 2021; Mathews et al. 2021; Wright et al. 2021).

In five studies, nurses reported perceiving that AD emphasises patient autonomy, which may at times overshadow other aspects of holistic care. Nurses report that once AD is requested, they perceive that the focus often shifts to procedural steps rather than the patient's overall needs, including their experiences, beliefs and social connections (Antonacci et al. 2021; Freeman et al. 2021; Ho, Norman, et al. 2021; Pesut, Thorne, Schiller, Greig, Roussel, and Tishelman 2020; Ward et al. 2022).

Studies that recruited their sample from a so‐called ‘non‐provider’ hospice setting indicate that nurses perceive that AD may fragment the patient‐provider relationship; this fragmentation often occurs when patients are transferred to a different setting for the AD procedure, leading nurses to report feelings of being ‘unfinished,’ ‘disrupted,’ or experiencing anticipated grief (Freeman et al. 2021; Ho, Norman, et al. 2021; Ward et al. 2022; Wright et al. 2021). Freeman et al. (2021) and Ward et al. (2022) reported that hospice nurses in this setting withdraw from patient relationships as a form of self‐protection, resulting in a sense of disconnection.

Participants in several studies expressed concern and sadness that patients may miss out on the full range of palliative support and reported questioning whether AD represents a failure of palliative care (Freeman et al. 2021; Ho, Norman, et al. 2021; Ward et al. 2022; Wright et al. 2021). In light of this, Ward et al. (2022, 11) concluded that ‘MAiD changed the dynamic and disrupted the ability of participants to provide “business as usual” care to patients.’

##### 4.2.3.2 Interacting With Relatives

Studies show that navigating complex family dynamics in the context of AD is a significant challenge for nurses (Freeman et al. 2021; Guité‐Verret et al. 2024; Ho, Joolaee, et al. 2021; Ho, Norman, et al. 2021; Mathews et al. 2021). Pesut, Thorne, et al. (2021) highlight that relatives of patients who request AD often find themselves in one of three positions: fully supportive, not reconciled but supportive or not reconciled and unsupportive. Nurses face particular challenges when dealing with families who are not supportive of the patient's decision, as they must navigate emotional tensions while maintaining a focus on the patient's wishes (Ho, Joolaee, et al. 2021; Ho, Norman, et al. 2021; Mathews et al. 2021). Ho, Norman, et al. (2021) point out that highly sensitive and complex situations occur when patients fear talking with their families about AD.

Furthermore, a study has shown that palliative care nurses who are excluded from discussions or decision‐making about AD feel awkward and uncertain about how to engage with families effectively (Freeman et al. 2021). In addition, a lack of clear information among relatives about the differences between palliative care and AD further complicates these interactions, as reported by Guité‐Verret et al. (2024) and Mathews et al. (2021).

Moreover, results from three studies highlight that nurses are concerned that grief following AD may manifest differently and highlight the need for tailored bereavement support, which emerges as a new challenge for nurses (Antonacci et al. 2021; Ho, Joolaee, et al. 2021; Pesut, Wright, et al. 2021).

##### 4.2.3.3 Collaboration

Several studies show that the diversity of opinions within teams regarding AD leads to conflicts and complicates collaboration (Buchbinder et al. 2019; Freeman et al. 2021; Ho, Joolaee, et al. 2021; Pesut, Thorne, et al. 2021; Ward et al. 2022; Wright et al. 2021). They indicate that professionals hesitate to express their views due to the fear of how colleagues might react (Pesut, Thorne, et al. 2021; Ward et al. 2022; Wright et al. 2021). This is particularly evident in team meetings ‘when dominant voices about MAiD, within palliative care, silence the potential for open dialogue and reflection’ (Wright et al. 2021, 8).

Furthermore, potential for conflict emerges from differing interpretations of institutional policies among team members (Buchbinder et al. 2019; Gerson, Preston, et al. 2020; Ho, Norman, et al. 2021; Joolaee et al. 2022; Pesut, Thorne, Schiller, Greig, and Roussel 2020; Pesut, Thorne, Schiller, Greig, Roussel, and Tishelman 2020; Pesut, Thorne, et al. 2021; Wright et al. 2021).

Two studies show that professionals from other disciplines often expect palliative care teams to take on the responsibility for AD, assuming they have the necessary competencies (Buchbinder et al. 2019; Mathews et al. 2021). Tensions arise when providers refuse to participate in AD, a position that can be difficult to understand for those outside of palliative care (Antonacci et al. 2021; Pesut, Thorne, Schiller, Greig, and Roussel 2020; Wright et al. 2021).

Despite these challenges, teamwork in the context of AD is described as highly valuable, particularly when respectful dialogue is possible and emotional tensions can be overcome (Joolaee et al. 2022; Ward et al. 2022).

## 5 Discussion

This review aimed to provide an overview of studies that examined impacts of the legalisation of AD on nursing practice in specialised palliative care. Palliative care is, by principle and according to major guidelines and international associations (e.g., WHO, EAPC), a setting in which AD is not part of routine practice. This approach allowed us to acknowledge that AD inevitably affects nursing practice more broadly, apart from focussing on direct involvement in the conduct of AD.

Our findings reveal multiple, complex impacts on nursing practice in specialised palliative care. Nurses face challenges in finding a clear professional position, as well as shifts in the scope of their core competencies, such as end‐of‐life conversations or symptom management. Although AD affects relational dynamics with patients, relatives and colleagues, nurses remain committed to maintaining caring relationships, going along with increased risks for moral distress. Beyond these practical challenges, the legalisation of AD prompts nurses to reflect on the broader societal meaning of palliative care.

All included studies originate from the North American context. We explored possible explanations for this concentration to better understand the contextual factors shaping research on AD. Other reviews also highlight the influence of regulatory frameworks on professional experiences of AD (Amankwaa et al. 2025; Klotz et al. 2025). In countries where nurses have no clearly defined role in AD processes or are not explicitly addressed in legal regulations, impacts on nursing practice may remain less visible and less likely to be studied. This effect may be intensified in palliative care nursing, as palliative care organisations in many countries oppose AD (Inbadas et al. 2020), which may discourage research on AD‐related impacts in this field. A delayed development of nursing science and limited research infrastructure compared to Canada or the US may also contribute to the absence of studies in some regions, for example in German‐speaking countries (Beil‐Hildebrand and Smith 2022). In addition, in several countries, including Austria (Unseld et al. 2025), Spain (Parra Jounou et al. 2024) and New Zealand (Wilson et al. 2019), AD has been legalised only recently. Although a few studies already exist, there has been limited time for systematic research, particularly on the impacts of AD on nursing practice in palliative care. By contrast, in the Switzerland, there is no specific legal regulation for AD (Gerson, Koksvik, et al. 2020) at all. In Switzerland, assisted suicide has long been addressed through a comparatively liberal criminal law approach (Feichtner and Wasl 2022). However, the absence of a specific statutory framework means that there has been no landmark legislative moment that might have stimulated research interest. From our perspective, the absence of studies beyond North America highlights an important research gap. This is increasingly relevant as more countries legalise AD, a development that is often accompanied by debates on its compatibility with the principles and boundaries of palliative care (Dorman et al. 2025). These developments are likely to place palliative care professionals and nurses in particular, at the centre of ethical challenges and moral questions that remain insufficiently explored.

Although palliative care and nursing are deeply intertwined, we found only one study focusing specifically on palliative care nurses (Wright et al. 2021). Notably, research studies tend to focus either on the palliative care context (Inbadas et al. 2020; Kitta et al. 2023) or on the nursing profession (Beuthin et al. 2018; Pesut et al. 2023) but rarely on their intersection. Additionally, our search revealed that many publications continue to explore nurses' general attitudes towards AD (Schroeder et al. 2024; Zeilinger et al. 2024). While understanding nurses' positions is important, our review also shows the need to move beyond attitudes and to put the focus on practical implications of AD legislations for nurses in palliative care.

Through our review, we focused on a group that is not directly involved in AD practice. The findings show that involvement in the relational and interactional aspects of care is difficult to delineate and often extends far beyond the procedural act itself. This aligns with recent evidence from Arshad et al. (2025), who showed that healthcare providers outside the formal AD process still play crucial roles. In practice, this blurred involvement becomes particularly evident in situations where nurses care for patients who express a wish for AD. Our review shows that such situations can challenge core competencies of nurses specialised in palliative care, such as conducting end‐of‐life conversations or managing symptoms appropriately. These disruptions may strain the quality of care and destabilise the professional identity of nurses in advanced practice roles like palliative care. In contrast, studies from broader nursing contexts have highlighted AD as an opportunity to develop advanced nursing roles and further strengthen nursing as a profession (Bustin et al. 2024). Furthermore, studies report about a sense of professional fulfilment when nurses can advocate for patient wishes regarding AD and support their right to self‐determination (Beuthin et al. 2018; Mills et al. 2023). Similar to the work of Dorman et al. (2025) who conducted an integrative review on the relationship between AD and palliative care, findings of our study suggest that AD may shift the focus away from holistic and relational models of care towards a more medicalised and procedural approach. This may, paradoxically, reduce nurses' autonomy, which is a central characteristic of advanced practice according to the definition of the International Council of Nurses (White et al. 2025). Such complexities suggest that further research is needed to better understand the interplay between nursing, palliative care and AD, and to advance a more nuanced and context‐sensitive research agenda.

At last, we would like to highlight our findings regarding nurses raising concerns about the redirection of already limited palliative care resources towards AD. Klotz et al. (2025) report, based on a systematic review, that AD requests can become morally burdensome when resources, particularly time, are scarce. Findings from the studies included in our review suggest that AD directly affects time resources within palliative care practice. This may contribute to a vicious circle in which increasing time pressures intensify moral distress among professionals. These concerns are grounded in the reality that access to palliative care is, and has long been, severely limited, with only a small proportion of those in need receiving appropriate services globally (Garralda et al. 2025). In this context, the legalisation of AD is not simply another end‐of‐life option, but it interacts with existing inequalities in care provision. Access becomes not merely a logistical or procedural matter, but a deeply moral question about how societies choose to allocate care, support and protection at the end of life (Pesut and Thorne 2025).

### 5.1 Strengths and Limitations

A strength of this review lies in its comprehensive focus on the impact of AD beyond nurses' direct involvement, thereby emphasising a broad nursing perspective. The exclusion of countries where AD has been integrated into palliative care since its early origins may have led to diverse experiences being overlooked. In addition, limiting the review to English and German publications and excluding grey literature may have contributed to the predominance of studies from a North American context, alongside the factors discussed above. Nevertheless, it has allowed us to specifically examine palliative care settings based on its principles.

Another limitation is the heterogeneity of the included studies. It was often difficult to define and compare levels of exposure to AD, a challenge that is common across research in this field and should be addressed in future studies to allow for more precise comparisons and stronger conclusions.

Finally, the analysis clearly focused on nursing perspectives. In cases where studies applied multiple datasets, shared multiprofessional findings were included if they also reflected nurses' perspectives. Although this approach was applied consistently, it cannot be fully excluded that the results were partly influenced by the perspectives of other professional groups.

## 6 Conclusions

The legalisation of AD significantly impacts nursing practice in palliative care. This development highlights the need for the nursing profession to engage in ethical reflection on its role and core values in this context. Palliative care nurses, with their in‐depth experience in end‐of‐life communication, ethical decision‐making and relational care, are particularly well equipped to contribute to this process. However, such reflection requires time, institutional support and protected spaces for dialogue and critical engagement.

Furthermore, the review shows the importance of differentiating between settings and professions in research on AD. Given the sensitivity and complexity of the topic, research in this field requires multiple perspectives, innovative methodologies and carefully formulated research questions. The perspective of palliative care nurses offers unique insights and adds important nuances to the broader evidence base on the challenges associated with the legalisation of AD.

## Funding

This review is part of the research programme of the Endowed Professorship for Nursing Science funded by Care with a Heart Foundation, Vienna, Austria.

## Conflicts of Interest

The authors declare no conflicts of interest.

## Supporting information


**Data S1:** jan70657‐sup‐0001‐Supinfo1.docx.

## Data Availability

The data that support the findings of this study are available from the corresponding author upon reasonable request.
