# Supplementary material for: The Impact of Assisted Dying Legislation on Nursing Practice in Palliative Care: A Scoping Review
Source: J Adv Nurs. 2026 Jun 5;82(12):10124–39. doi: 10.1111/jan.70657 (PMC13752660; doi:10.1111/jan.70657)
Supplement: Supplementary file 1 [file JAN-82-10124-s001.docx]

**Search History: Impact of assisted dying on nursing practice in palliative care (CENTRAL)**
Search performed on 1.8.2024

| **#** | **Query** | **Results from 1 Aug 2024** |
| --- | --- | --- |
| 1 | exp "Hospice and Palliative Care Nursing"/ | 97 |
| 2 | exp Nursing/ | 4,330 |
| 3 | exp Nurses/ | 1,754 |
| 4 | exp nursing staff/ | 852 |
| 5 | exp Nurse's Role/ | 617 |
| 6 | 2 or 3 or 4 or 5 | 6,669 |
| 7 | exp Palliative Care/ | 2,559 |
| 8 | exp Hospice Care/ | 179 |
| 9 | 7 or 8 | 2,684 |
| 10 | 6 and 9 | 151 |
| 11 | (nurs* and (palliat* or hospice* or "end of life")).ti,ab,kf. | 1,324 |
| 12 | 1 or 10 or 11 | 1,419 |
| 13 | exp Suicide, Assisted/ | 10 |
| 14 | exp Euthanasia/ not Euthanasia, Animal/ | 12 |
| 15 | ("assisted suicide*" or "assisted death*" or "assisted dying" or "assistance in dying" or "aid in dying").ti,ab,kf. | 15 |
| 16 | (euthan* or "mercy killing*").ti,ab,kf. | 239 |
| 17 | ("death with dignity" or "hastened death*" or "hastening death*" or "hasten death*").ti,ab,kf. | 23 |
| 18 | 13 or 14 or 15 or 16 or 17 | 277 |
| 19 | 12 and 18 | 3 |

**Search History: Impact of assisted dying on nursing practice in palliative care (CINAHL)**
Search performed on.7.2024

| **#** | **Query** | **R**esults |
| --- | --- | --- |
| S31 | S29 AND S30 | **687** |
| S30 | LA (English OR German) | 8,627,755 |
| S29 | S27 NOT S28 | 708 |
| S28 | PT (Commentary OR Editorial) | 621,058 |
| S27 | S23 NOT S26 | 748 |
| S26 | S24 NOT S25 | 725,347 |
| S25 | AG All Adult | 2,030,086 |
| S24 | AG (All Infant OR All Child) | 1,127,308 |
| S23 | S16 AND S22 | 778 |
| S22 | S17 OR S18 OR S19 OR S20 OR S21 | 15,038 |
| S21 | TI ( "death with dignity" OR "hastened death*" OR "hastening death*" OR "hasten death*" ) OR AB ( "death with dignity" OR "hastened death*" OR "hastening death*" OR "hasten death*" ) | 738 |
| S20 | TI ( euthan* OR "mercy killing*" ) OR AB ( euthan* OR "mercy killing*" ) | 5,332 |
| S19 | TI ( "assisted suicide*" OR "assisted death*" OR "assisted dying" OR "assistance in dying" OR "aid in dying" ) OR AB ( "assisted suicide*" OR "assisted death*" OR "assisted dying" OR "assistance in dying" OR "aid in dying" ) | 3,287 |
| S18 | (MH "Euthanasia+") | 8,398 |
| S17 | (MH "Suicide, Assisted") | 4,160 |
| S16 | S5 OR S14 OR S15 | 19,630 |
| S15 | TI ( (nurs*) AND (palliat* OR hospice* OR "end of life") ) OR AB ( (nurs*) AND (palliat* OR hospice* OR "end of life") ) | 13,913 |
| S14 | S10 AND S13 | 6,641 |
| S13 | S11 OR S12 | 50,484 |
| S12 | (MH "Hospice Care") | 10,057 |
| S11 | (MH "Palliative Care") | 43,672 |
| S10 | S6 OR S7 OR S8 OR S9 | 536,797 |
| S9 | (MH "Nursing Role") | 63,912 |
| S8 | (MH "Nurses+") | 236,133 |
| S7 | (MH "Nursing as a Profession+") | 279,081 |
| S6 | (MH "Nursing Care+") | 305,324 |
| S5 | S1 OR S2 OR S3 OR S4 | 5,895 |
| S4 | (MH "Hospice Nurses") | 67 |
| S3 | (MH "Hospice Nursing") | 5,509 |
| S2 | (MH "Palliative Care Nurses") | 155 |
| S1 | (MH "Palliative Care Nursing") | 281 |

**Search History: Impact of assisted dying on nursing practice in palliative care (Embase)**
Search performed on 1.8.2024

| **No.** | **Query** | **Results** |
| --- | --- | --- |
| #27 | #25 AND #26 | 653 |
| #26 | english:la OR german:la | 40223242 |
| #25 | #23 NOT #24 | 694 |
| #24 | editorial:it OR comment:it | 808411 |
| #23 | #21 NOT #22 | 705 |
| #22 | 'child'/exp NOT ('adolescent'/exp OR 'adult'/exp) | 2076852 |
| #21 | #14 AND #20 | 723 |
| #20 | #15 OR #16 OR #17 OR #18 OR #19 | 71800 |
| #19 | 'death with dignity':ti,ab,kw OR 'hastened death*':ti,ab,kw OR 'hastening death*':ti,ab,kw OR 'hasten death*':ti,ab,kw | 1536 |
| #18 | euthan*:ti,ab,kw OR 'mercy killing*':ti,ab,kw | 55903 |
| #17 | 'assisted suicide*':ti,ab,kw OR 'assisted death*':ti,ab,kw OR 'assisted dying':ti,ab,kw OR 'assistance in dying':ti,ab,kw OR 'aid in dying':ti,ab,kw | 6011 |
| #16 | 'euthanasia'/exp NOT 'animal euthanasia'/de | [24831](https://www.embase.com/) |
| #15 | 'assisted suicide'/exp | 5909 |
| #14 | #1 OR #10 OR #13 | 30176 |
| #13 | #11 AND #12 | 24076 |
| #12 | palliat*:ti,ab,kw OR hospice*:ti,ab,kw OR 'end of life':ti,ab,kw | 204083 |
| #11 | nurs*:ti,ab,kw | 674069 |
| #10 | #6 AND #9 | 15038 |
| #9 | #7 OR #8 | 160313 |
| #8 | 'hospice care'/exp | 14193 |
| #7 | 'palliative therapy'/exp | 151916 |
| #6 | #2 OR #3 OR #4 OR #5 | 667210 |
| #5 | 'nurse attitude'/exp | 44341 |
| #4 | 'nursing staff'/exp | 79419 |
| #3 | 'nurse'/exp | 230970 |
| #2 | 'nursing'/exp | 429341 |
| #1 | 'palliative nursing'/exp | 1788 |

**Search History: Impact of assisted dying on nursing practice in palliative care (PsycInfo)**
Search performed on 31.7.2024

| **#** | **Query** | **Results** |
| --- | --- | --- |
| 1 | (nurs* and (palliat* or hospice* or "end of life")).ti,ab,hw,id,mf. | 5915 |
| 2 | ("assisted suicide*" or "assisted death*" or "assisted dying" or "assistance in dying" or "aid in dying").ti,ab,hw,id,mf. | 2116 |
| 3 | (euthan* or "mercy killing*").ti,ab,hw,id,mf. | 3546 |
| 4 | ("death with dignity" or "hastened death*" or "hastening death*" or "hasten death*").ti,ab,hw,id,mf. | 566 |
| 5 | 2 or 3 or 4 | 4757 |
| 6 | 1 and 5 | 234 |
| 7 | limit 6 to (english or german) | **230** |

**Search History: Impact of assisted dying on nursing practice in palliative care (PubMed)**
Search performed on 31.7.2024

| **#** | **Query** | **Results** |
| --- | --- | --- |
| 17 | #15 AND #16 | **684** |
| 16 | english[la] OR german[la] | 33,600,085 |
| 15 | #13 NOT #14 | 714 |
| 14 | comment[pt] OR editorial[pt] | 1,525,750 |
| 13 | #11 NOT #12 | 738 |
| 12 | ((infant[mesh] OR child[mesh]) NOT (adolescent[mesh] OR adult[mesh])) | 1,503,754 |
| 11 | #4 AND #10 | 759 |
| 10 | #5 OR #6 OR #7 OR #8 OR #9 | 56,303 |
| 9 | "death with dignity"[Title/Abstract] OR "hastened death*"[Title/Abstract] OR "hastening death*"[Title/Abstract] OR "hasten death*"[Title/Abstract] | 1,352 |
| 8 | euthan*[Title/Abstract] OR "mercy killing*"[Title/Abstract] | 47,857 |
| 7 | "assisted suicide*"[Title/Abstract] OR "assisted death*"[Title/Abstract] OR "assisted dying"[Title/Abstract] OR "assistance in dying"[Title/Abstract] OR "aid in dying"[Title/Abstract] | 5,330 |
| 6 | euthanasia[mesh] NOT euthanasia, animal[mesh] | 13,955 |
| 5 | suicide, assisted[mesh] | 6,514 |
| 4 | #1 OR #2 OR #3 | 18,033 |
| 3 | (nurs*[Title/Abstract]) AND (palliat*[Title/Abstract] OR hospice*[Title/Abstract] OR "end of life"[Title/Abstract]) | 14,737 |
| 2 | (nursing[mesh] OR nurses[mesh] OR nursing staff[mesh] OR nurse's role[mesh]) AND (palliative care[mesh] OR hospice care[mesh]) | 5,525 |
| 1 | Hospice and Palliative Care Nursing[mesh] | 2,571 |

**Search String:**

((((((Hospice and Palliative Care Nursing[mesh]) OR ((nursing[mesh] OR nurses[mesh] OR nursing staff[mesh] OR nurse's role[mesh]) AND (palliative care[mesh] OR hospice care[mesh])) OR ((nurs*[Title/Abstract]) AND (palliat*[Title/Abstract] OR hospice*[Title/Abstract] OR "end of life"[Title/Abstract]))) AND ((suicide, assisted[mesh]) OR (euthanasia[mesh] NOT euthanasia, animal[mesh]) OR ("assisted suicide*"[Title/Abstract] OR "assisted death*"[Title/Abstract] OR "assisted dying"[Title/Abstract] OR "assistance in dying"[Title/Abstract] OR "aid in dying"[Title/Abstract]) OR (euthan*[Title/Abstract] OR "mercy killing*"[Title/Abstract]) OR ("death with dignity"[Title/Abstract] OR "hastened death*"[Title/Abstract] OR "hastening death*"[Title/Abstract] OR "hasten death*"[Title/Abstract]))) NOT (((infant[mesh] OR child[mesh]) NOT (adolescent[mesh] OR adult[mesh])))) NOT (comment[pt] OR editorial[pt])) AND (english[la] OR german[la]))
